# Imaging of Bone in the Head and Neck Region, is There More Than CT?

**DOI:** 10.1007/s40134-022-00396-8

**Published:** 2022-04-16

**Authors:** Karen A. Eley, Gaspar Delso

**Affiliations:** 1 Department of Radiology, School of Clinical Medicine, University of Cambridge, Cambridge Biomedical Campus, Box 218, Cambridge, CB2 0QQ UK; 2 MR Applications & Workflow, GE Healthcare, Barcelona, Spain

**Keywords:** MRI, Bone, Craniofacial, Imaging, Radiation protection, 3D imaging

## Abstract

**Purpose of Review:**

The objective of this review is to document the advances in non-ionising imaging alternatives to CT for the head and neck.

**Recent Findings:**

The main alternative to CT for imaging bone of the head and neck region is MRI, particularly techniques which incorporate gradient echo imaging (Black Bone technique) and ultra-short or zero-echo time imaging. Since these techniques can provide high resolution isometric voxels, they can be used to provide multi-planar reformats and, following post processing, 3D reconstructed images of the craniofacial skeleton. As expected, the greatest advancements in recent years have been focused on enhanced image processing techniques and attempts to address the difficulties encountered at air-bone interfaces.

**Summary:**

This article will review the imaging techniques and recent advancements which are bringing non-ionising alternatives to CT imaging of the bone of the head and neck region into the realm of routine clinical application.

## Introduction

Imaging the head and neck is notoriously depicted as being complex. It requires a thorough understanding of anatomy, which includes the many crevices and foramen of the craniofacial skeleton. It is therefore understandable that CT, with its rapid acquisition and ease with which bone can be segmented to produce 3D rendered images, has become the staple imaging modality for both benign and malignant pathologies. However, as CT has become widespread so has recognition of the potential long-term harms of repeated ionising radiation exposure, particularly in young patients with benign conditions. The head and neck region is particularly vulnerable due to the presence of radiosensitive tissues such as the thyroid and the lens of the eye.

Hounsfield, in his 1973 paper on CT, stated that “the exposure of the patient to x-rays must be restricted” [1]. Currently, in the 50th anniversary year of the first commercially viable CT scanner, it is pertinent to re-evaluate if there is more than just CT to image bones of the head and neck region.

## Why Seek Alternatives to CT

Whilst recent developments in CT technology have seen a reduction in ionising radiation dose, the potential deleterious effects of cumulative ionising radiation exposure remain a concern. Repeated exposure to head and neck CT, for example, is significantly associated with increased risk of cataract [2]. Young patients pose the greatest risk in terms of exposure, with an estimated risk in one study for the 10 years following CT exposure equating to one brain tumour per 10,000 patients exposed to a 10 mGy scan at less than 10 years of age [3]. Dremmen et al. [4] highlighted that whilst the lifetime cancer mortality risk attributable to the radiation from a single CT head scan is low (approximately 0.07% in a 1-year-old child), the frequency with which head trauma occurs in this population translates to a large population-level risk. Added to this is the increasing utility of ionising radiation in the form of cone-beam CT (CBCT); CBCT is now frequently employed in the outpatient setting (including dentists) where previously a low-dose radiograph would have sufficed. The effective dose from CBCT can vary widely across machines and reach levels equivalent to that of conventional CT [5].

CT can be rapidly acquired and provides high resolution bony imaging capable of multi-directional and 3D reformatted images. It is infrequently affected by motion artefacts, unlike its cross-sectional counterpart, MRI. MRI, however, offers exquisite soft tissue detail of the head and neck, providing enhanced characterisation of pathology. As a result, MRI is increasingly utilised for radiotherapy planning for head and neck cancer, helping to reduce systematic MRI-CT co-registration errors, medical cost, radiation exposure and simplifying the clinical workflow [6].

The main challenge of MRI bone imaging, compared to CT, is that MR images cannot be directly related to tissue density. This is because MRI voxel intensity is related to proton density (affected by the magnetic properties of tissue) rather than electron density, and a one-to-one relationship between them does not exist [7]. This is why attenuation correction in PET-MR imaging is problematic.

As a result, multi-modality imaging is frequently employed with many patients undergoing CT, MRI and ultrasound examinations when imaging the head and neck. Over the past 18 months, the SARS-Cov2 pandemic has placed further demand on radiology services. Being able to eliminate additional investigations would be beneficial in terms of cost, departmental efficiency, and patient satisfaction.

## Alternatives to CT

The two main alternatives to CT to consider for imaging bone of the head and neck region are ultrasound and MRI. Other techniques such as optical coherence tomography which can evaluate the microstructure of bone, are beyond the scope of this review.

### Ultrasound

Ultrasound provides excellent soft tissue imaging of the head and neck. Whilst it is not typically the modality of choice to evaluate bony structures, the cortical bone surface is easily delineated, making superficial pathologies such as osteomas easy to diagnose. Cranial ultrasound is frequently employed in the paediatric setting, permitting evaluation of bone, cranial sutures and intracranial contents.

#### Cranial Sutures

In infants, cranial ultrasound provides both excellent visualisation of bony pathologies with intracranial assessment being afforded by patent fontanelles and often minimal hair. In the investigation of abnormal head shape in infants, ultrasound has been shown to offer comparable accuracy to CT in the diagnosis of craniosynostosis. Rozovsky et al. [8] evaluated the sensitivity and specificity of ultrasound compared to radiography in infants investigated for main sutural craniosynostosis. Ultrasound performed comparably to radiography with a sensitivity of 100% and specificity of 98%. Discrepancy occurred in two infants for the metopic suture in whom physiological closure of the suture had taken place, being mistaken for synostosis on ultrasound. Interpreting the ultrasound findings in unison with the clinical appearances would have avoided this diagnostic error—the absence of trigonocephaly providing significant reassurance that the suture was not prematurely fused. Similar results were reported by Alizadeh et al. [9] in their study of 44 infants under the age of 1 year with clinical suspicion of craniosynostosis. Sensitivity and specificity of ultrasound was 96.9% and 100% respectively.

The main difficulty associated with using ultrasound for diagnosis of craniosynostosis is the inability to produce 3D reconstructed imaging of the whole cranium, which many surgeons require for surgical planning.

#### Trauma

In the investigation of traumatic skull fractures, Rabiner et al. [10] demonstrated a sensitivity of 88% and specificity of 97% in a cohort of 69 patients with a mean age of 6.4 years. Similar results were obtained by Parri et al. [11] in a cohort of 115 patients under the age of 2 years.

The evidence for point of care ultrasound for the diagnosis of skull fractures in children has been recently reviewed in a systematic review and meta-analysis by Alexandridis et al. [12* *], who appraised 7 relevant studies. Sensitivity ranged from 67–100% and specificity from 85%-100%. Similar findings were reported in a further systematic review by Gordon et al. [13].

Good intracranial views can often be achieved with ultrasound, particularly during the neonatal period, which permits completion of the examination. Masaeli et al. [14] compared the ultrasound and CT findings of 538 patients under the age of 18 years. The point of care ultrasound was performed by attending emergency physicians and third year residents with a sensitivity of 85.7% and specificity of 97.9% for the detection of intracranial haemorrhage in children under 2 years of age, with good concordance with CT findings. There was however 1 false negative and 3 false positives in this age group.

In the trauma setting, the ability to perform bedside ultrasound in the emergency department, is highly beneficial, offering a rapid mode of assessment, without the need for ionising radiation. Consideration does however need to be given to the mechanism of injury and the potential of abusive head trauma (non-accidental injury) which will necessitate full investigation with cross-sectional imaging to fully document all injuries.

As with all ultrasound examinations, there is reliance upon operator expertise, with the potential for misinterpretation, patient non-compliance in the paediatric setting and the inability to provide a clear surgical roadmap for the surgeon. Increased reliance is placed on the ultrasound report due to unfamiliarity of ultrasound images by the clinical team, and is notoriously disliked for this reason. Frequently, point of care ultrasounds are performed by the resident clinical team, with wide variability in ultrasound experience and expertise. In the meta-analysis by Alexandridis et al. [12* *], ultrasounds were typically performed by clinicians after a training period of 1 h. This will contribute to the reported specificity of the various studies, with operators with the most experience and training invariably attaining the most accurate diagnoses.

### MRI

The main contender for bone imaging therefore lies with MRI, which will be the focus for the remainder of this review. In recent years techniques have developed to offer volumetric acquisition with isometric voxels. However, MR depiction of solid bone structures is challenging due to low proton density (~ 20% of water) and heterogeneous structure, which leads to short signal lifetimes [15]. Techniques to enhance bony detail and create 3D rendered images is an area of ongoing research, in which considerable progress has been achieved over the past 14 years.

#### MRI Bone Techniques

##### Black Bone (GRE-BB)

Tissues such as cortical bone have tightly bound protons, resulting in local magnetic field heterogeneity and short spin–spin (T2) relaxation times (< 1 ms), leading to weak MR signal which decays very rapidly. The relatively long echo times (TE) used in conventional imaging will produce little or no signal and appear dark (or “black”). Whilst tissues such as cortical bone, tendons, ligaments, and menisci contain a majority of short T2 components, other tissues also contain short T2 relaxation components, but as a minority species [16].

**Fig. 1:**
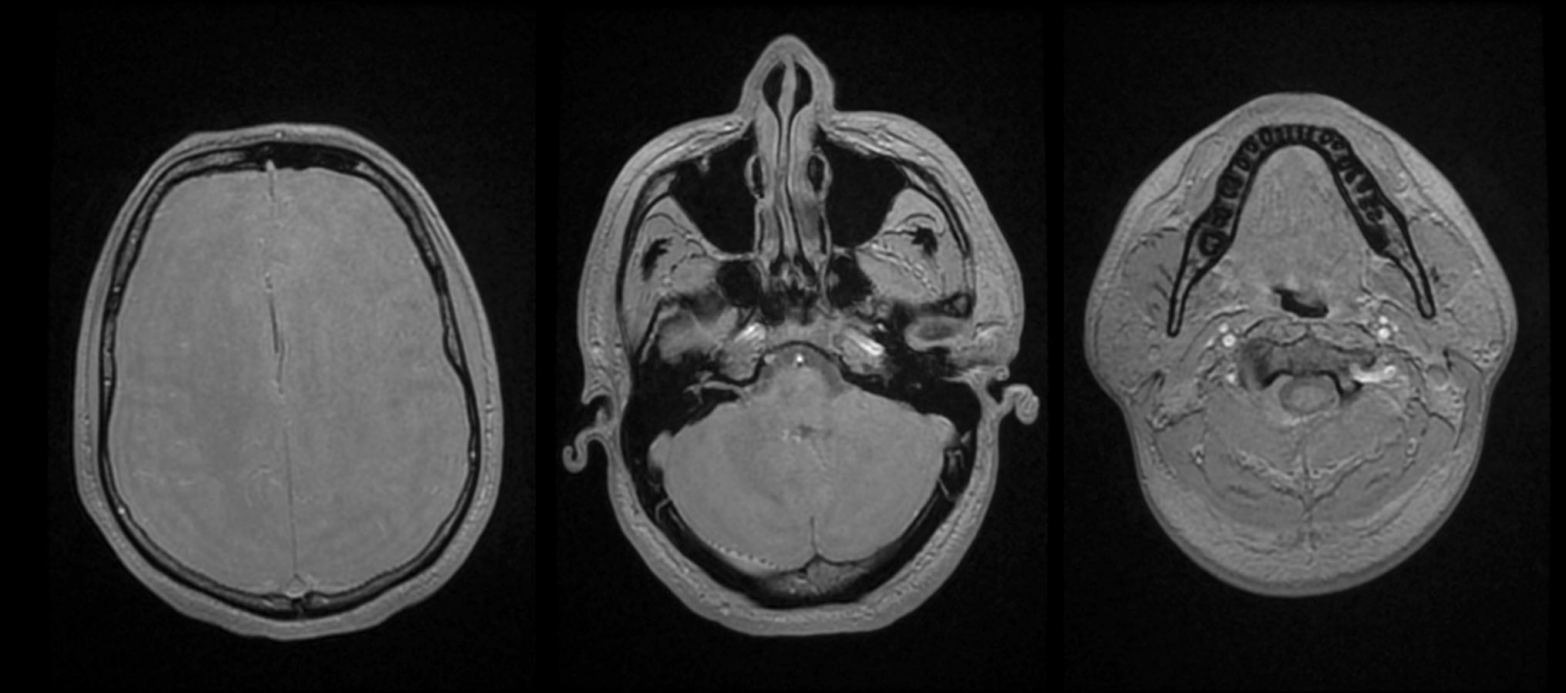
Axial GRE-BB imaging from an adult volunteer demonstrating uniform soft tissue contrast and “black” bone

The first reported MRI technique in the literature for imaging bone of the craniofacial region was referred to as “Black Bone”, acknowledging this short T2 effect. By manipulating imaging parameters to make soft tissue intensities as uniform as possible (i.e., approaching proton-density weighting), the bone soft-tissue interface is enhanced [17] (Fig. 1). The initial descriptions of Black Bone utilised a gradient echo sequence (GRE-BB), with short TE, short TR and a flip angle of 5 degrees. With a 3D volume acquisition, a voxel size of approximately 1.2 mm is achieved with a scan time in the region of 5 min for an adult head. Biometric accuracy has been confirmed through phantom work [17]. Whilst the initial imaging was performed using GE scanners, translation to other vendors produced similar results with Philips and Siemens MR systems [18**, 19]. The technique relies on a gradient echo (GRE) sequence such as 3D fast GRE (GE Medical Systems Ltd, Chalfont St Giles, UK), volumetric interpolated breath-hold examination (Siemens Healthcare Ltd, Camberley, UK) or an equivalent *T*_1_ weighted GRE 3D volume sequence (Philips Healthcare, Guildford, UK). The main limitation of the technique is in areas where air and bone come into contact, since the two species essentially yield no signal and therefore cannot be distinguished. The main advantage, however, is that this simple technique can be performed on all magnets without requiring any additional sequences or hardware.

Over recent years, the term Black Bone has been used interchangeably for a variety of MRI techniques whereby the goal remains the same, yet the fundamental physics can be considerably different.

##### UTE/ZTE

A family of clinically compatible sequences is capable of achieving TE values less than 1 ms, including ultrashort echo time (UTE) and zero echo time (ZTE) sequences [20]. In the case of UTE, short excitation pulses followed by radial readout are used to minimise the time between excitation and the acquisition of the k-space centre. This results in echo times of a few tens of microseconds, capable of recording the rapidly fading signal from cortical bone. One important advantage of detecting bone signal, however weak, comes for automated segmentation. Indeed, by combining two or more acquisitions with different echo times, it is possible to identify and separate long T2 species from cartilage and bone.

A further reduction of the dead time between excitation and readout can be achieved by ramping up the encoding gradients before, rather than after, the excitation pulse. With this approach, used in sequences such as Pointwise Encoding Time Reduction with Radial Acquisition (PETRA) and Zero Echo Time (ZTE), echo times are mainly limited by the hardware’s ability to switch between transmit and receive modes. The high sampling efficiency of close to 100% (i.e., most of the TR is used to acquire data) provides efficient scanning and offers a flexible trade-off between bandwidth, number of averages and signal-to-noise ratio [15]. Indeed, the signal-to-noise improvement brought by the shorter echo times enables the segmentation of bone structures from a single acquisition, with the subsequent time gain with respect to dual-echo UTE approaches.

**Fig. 2:**
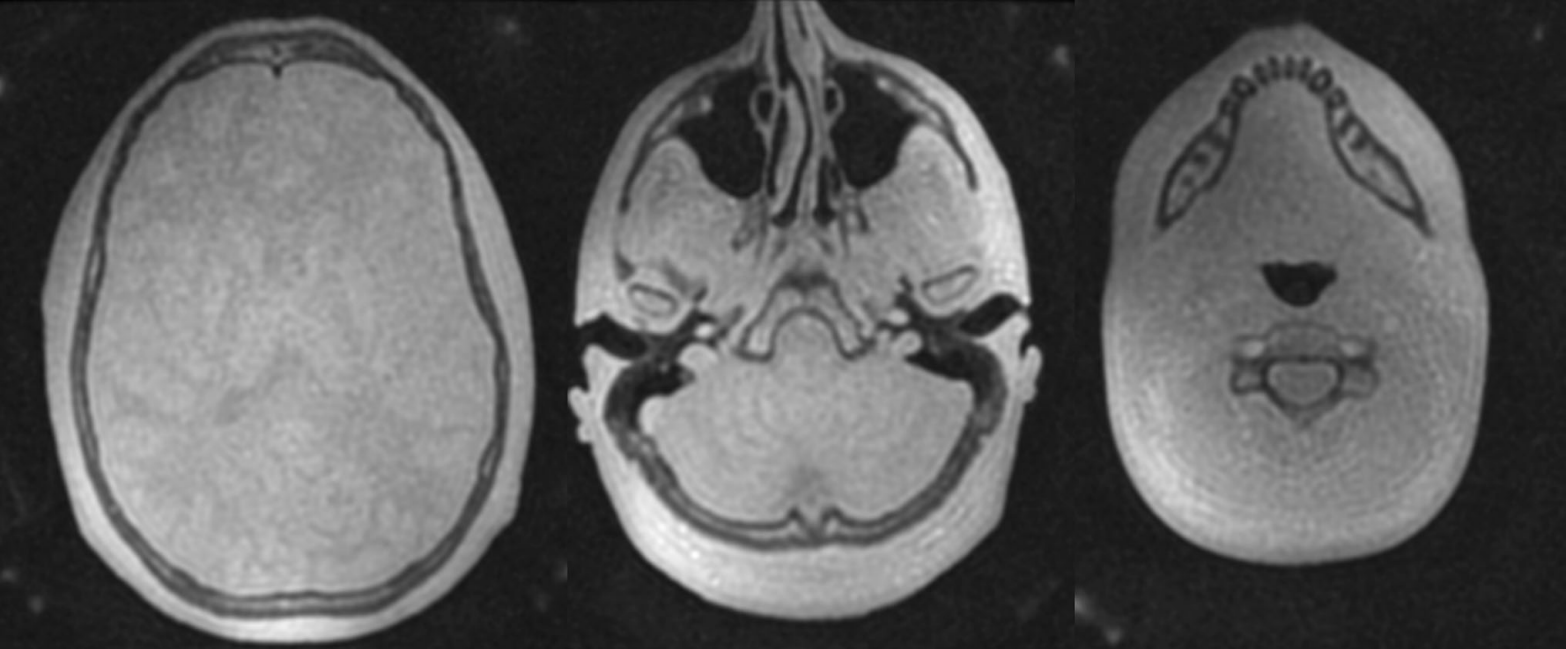
Axial ZTE imaging from an adult volunteer. Note the lower resolution of imaging (compared to GRE-BB) which can be acquired in 1 min 16 s (compared to 4 min 48 s)

As a 3D acquisition, these sequences can obtain isotropic resolution with 1 mm slice thickness, with an acquisition time of approximately 4 min for the head (Fig. 2). A significant impetus for the development of ZTE sequences was for attenuation correction in PET/MRI, but ZTE also shows considerable potential in imaging a range of bony pathology.

Conventional MRI sequences result in acoustic noise in the region of 90–130 dBA due to Lorentz forces caused by rapidly changing currents in the magnetic field gradient coils primarily used for spatial localisation [21]. This is a significant contributing factor in failed MRI examinations, particularly in the paediatric population (where imaging during natural sleep is often attempted), with resultant motion artefacts or premature termination of the study. However, with ZTE, gradient switching can be reduced to a minimum which enables quiet operation, being highly beneficial for paediatric imaging [21]. The main limitation of ZTE techniques is the hardware requirements, such as high-performance coils permitting rapid switching between transmit and receive. As such, UTE/ZTE cannot be adopted on all scanners. 

##### CT-like and Synthetic or Pseudo-CT Imaging

**Fig. 3:**
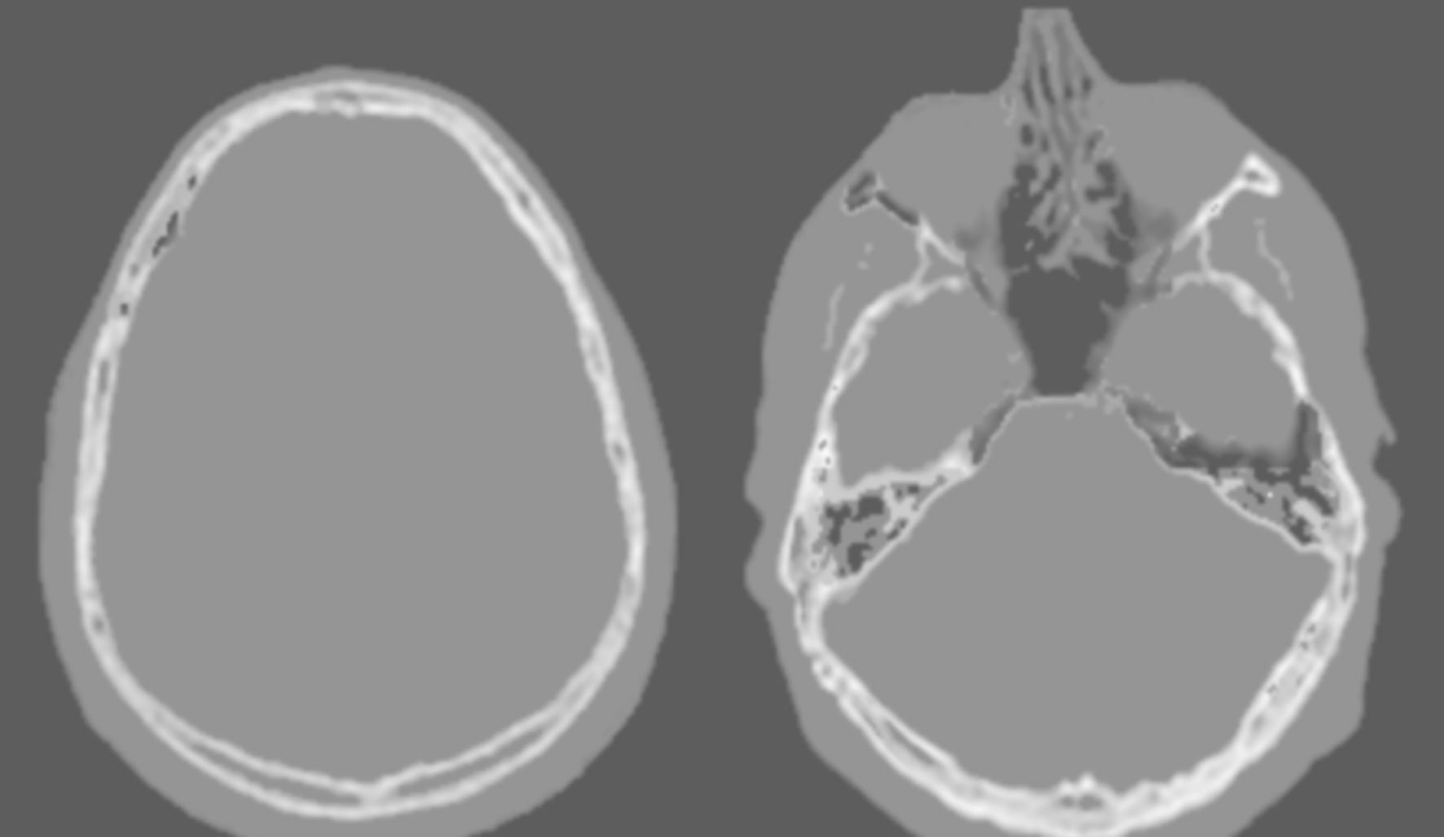
Examples of post-processed pseudo—CT images created from ZTE imaging

Various techniques have been utilised to generate CT-like images from MRI datasets (Fig. 3). Two broad sub-types exist – those that mimic the visual appearance of CT images and quantitative synthetic CT images that are based on the accurate approximation of measurable Hounsfield units [22]. Utilising a deep learning-based multiparametric MRI technique, Jans et al. [23] were able to create synthetic CT images that qualitatively and quantitatively resemble conventional bone window CT images.

#### 3D Reconstruction Techniques

##### Black Bone

**Fig. 4:**
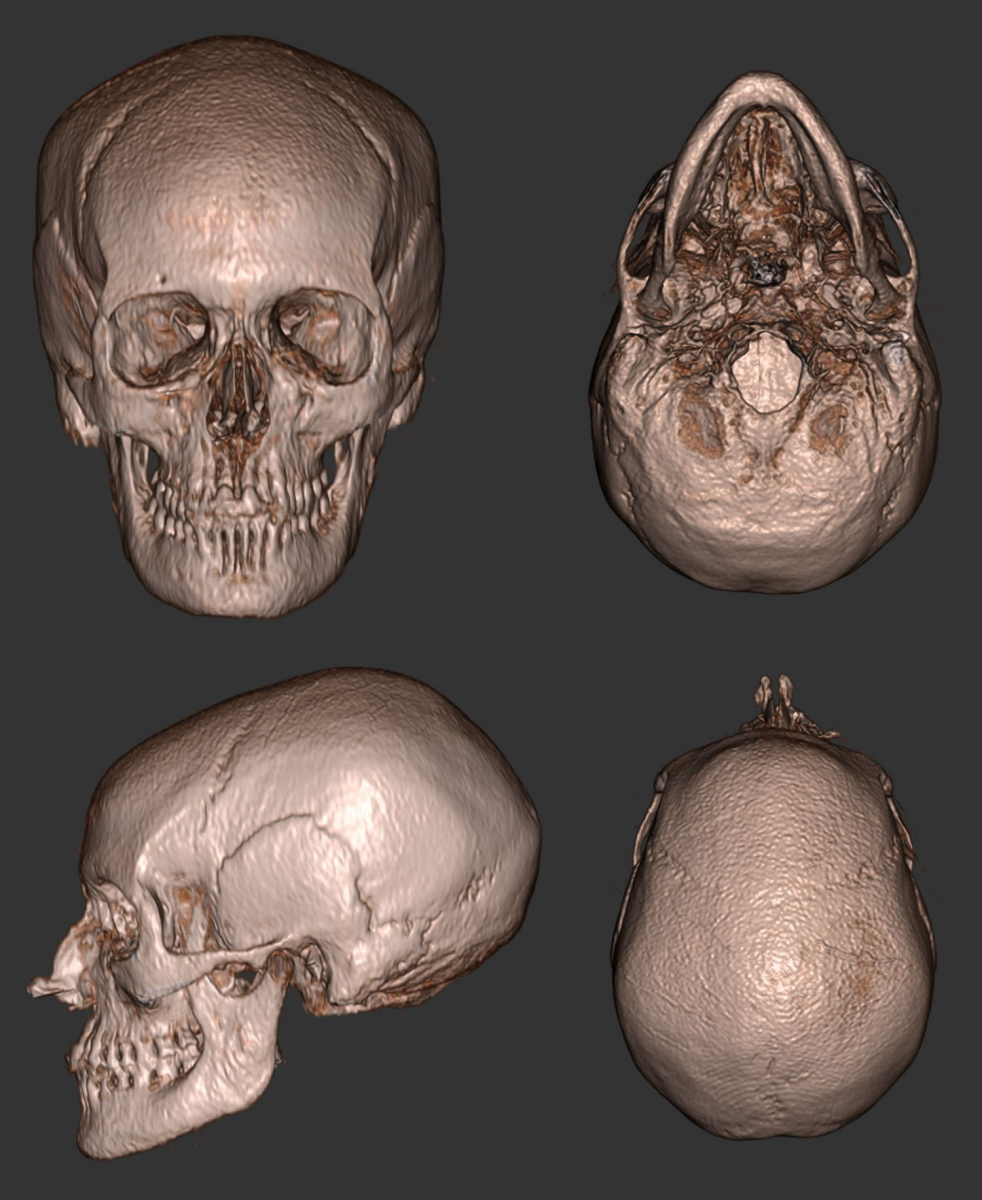
3D reconstructed GRE-BB imaging from an adult volunteer. The post-processed imaging was imported into Fovia High-definition volume rendering software to achieve excellent 3D results

The first reports of 3D reconstruction of the craniofacial skeleton from GRE-BB were presented at the CARS conference in 2011 [24], with subsequent publications in 2014 focused on children with craniosynostosis [18**]. The GRE-BB sequence was successfully utilised to 3D render the craniofacial skeleton using Fovia High-Definition volume rendering software (Fovia Inc, Palo Alto, CA, USA). This necessitated the selection of an appropriate transfer function, whereby a colour and opacity were applied to every voxel, followed by segmentation to remove the soft tissues. Even with some semi-automated processing, the average time for image processing was in the region of 13 min per dataset in experienced hands. Further results utilising mainstream software such as Mimics (Materialise, Leuven, Belgium), and Osirix (Pixmeo, Switzerland) were also described, with varying results [25]. Fully automated segmentation algorithms have now been achieved for adult GRE-BB datasets, with ongoing work to develop similar results in young infants [26] (Fig. 4). For automated segmentation, a series of steps are required to produce pseudo-CT images with the bone appearing hyperintense in comparison to surrounding tissues. It is not possible to simply invert the image to achieve this, since air and bone have similar pixel values, and thus the head would be obscured by a box of hyperintense air included in the field of view.

##### ZTE

In 2016, Wiesinger et al. [15] demonstrated the potential of ZTE for 3D reconstruction of the skull. The high resolution ZTE sequence took 6 min 12 s to acquire images of the head in their adult volunteers. Following bias correction and signal normalisation, the images were segmented into three classes: air, soft tissue and bone, and the pseudo-CT imaging successfully 3D rendered. Lu et al. [27] imaged 14 patients (2 months to 17 years of age) with ZTE for a range of clinical indications, the majority of which were skull deformity either congenital or traumatic. Most patients were imaged with both CT and ZTE, and the authors reported similar success with 3D rendering.

##### Other MRI Techniques

Maeda et al. [28] utilised a 3D T1-weighted sequence to surface render the mandible, and compared the results to CT. They reported a 3D absolute difference between CT and MRI of 1.6 mm. The use of a T1-weighted sequence will have contributed to this discrepancy, in view of the greater challenges and reliance upon manual segmentation for a non-bone specific MRI technique.

##### Combining MRI Techniques

**Fig. 5:**
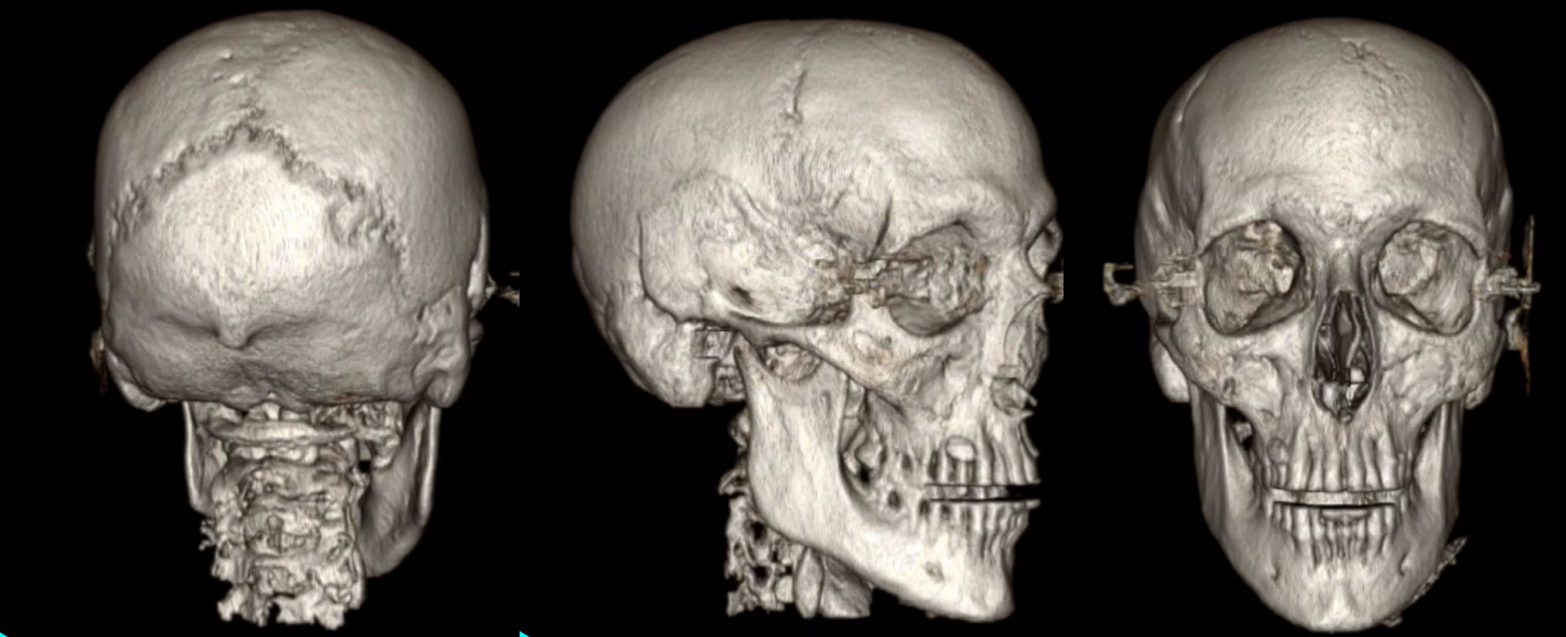
Automated 3D rendered FIESTA-C imaging using ZTE to drive the segmentation process

By utilising the main advantage of ZTE, namely that it is the optimal method to discern air from bone and using it with another sequence such as GRE-BB, to provide high resolution imaging without the inefficiency of radial sampling, 3D segmentation algorithms are further enhanced [29*]. This helps to minimise non-bone containing structures, such as ligamentous attachments as well as air. These technical developments, utilising ZTE as the driver, also permits the use of alternative MRI sequences, such as FIESTA-C (fast imaging employing steady-state acquisition; GE Healthcare), which offers high resolution imaging but with slightly shorter acquisition times (Fig. 5). FIESTA-C is used more routinely in clinical practice, and thus widens the scope of clinical utility, since only the relatively rapid acquisition of ZTE imaging would be required to permit 3D bone reconstruction; low-resolution ZTE imaging of the head being acquired in little over one minute.

Utilising FIESTA-C imaging to produce 3D images of the skull, and time of flight imaging of vessels Hayashi et al. [30] were able to successfully create 3D visualisation of the frontal and parietal branches of the superficial temporal artery, in patients undergoing investigation for ICA-ECA bypass. This provided superior evaluation of the vessels than CT due to beam hardening of the skull.

#### Clinical Applications

Bone-specific MRI techniques have largely been focused on paediatric pathology in view of the greatest potential in terms of radiation protection.

##### Cranial Sutures and Craniosynostosis

**Fig. 6:**
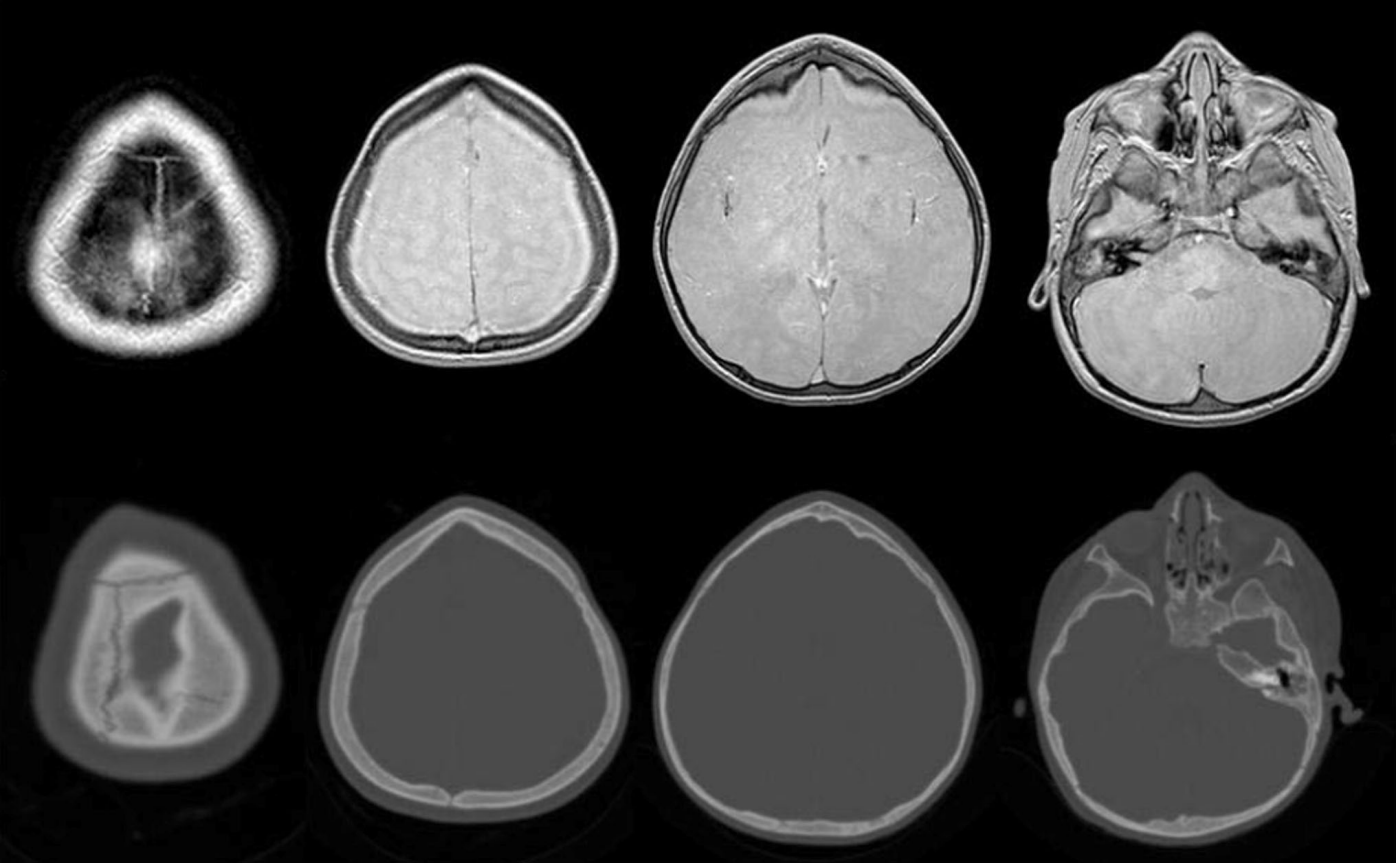
Axial GRE-BB (top row) and CT (bottom row) in a 17-month-old child with metopic synostosis. Note the trigonocephaly and absence of metopic suture, but the normal coronal sutures

**Fig. 7:**
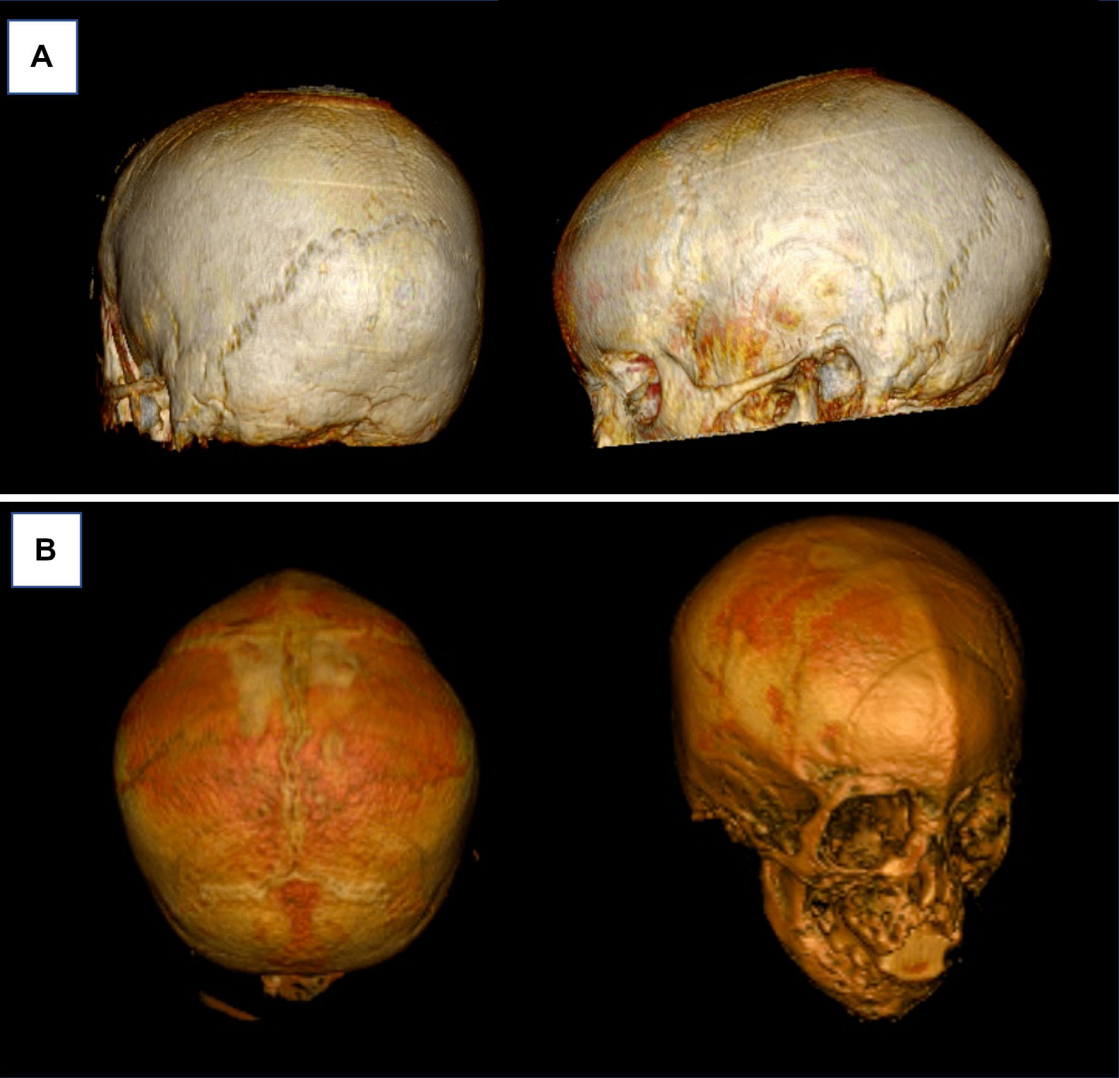
Semi-automated 3D rendering of GRE-BB datasets in a child with sagittal synostosis (**A**), and metopic synostosis (**B**). Note that the normal patent sutures are clearly visualised distinct from those that are prematurely fused. There is a ridge associated with the metopic synostosis

The earliest work in this area was in children with craniosynostosis. GRE-BB successfully depicted the normal cranial suture as an area of hyperintense signal, distinct from those which were prematurely fused and thus not visualised [18**] (Fig. 6). GRE-BB offers enhanced detection of the unfused cranial sutures to ZTE. Cranial suture detection was further enhanced by the 3D reconstruction work previously discussed [18**]. Fully automated segmentation algorithms have recently been applied to these datasets with variable results (Fig. 7). Ongoing research in this area is likely to yield a fully automated segmentation for young infants. Patel et al. [31] utilised a Siemens GE-VIBE (fast low-angle shot golden-angle 3D stack-of-stars radial VIBE) base sequence on a 3 T magnet, with similar TR/TE and a flip angle of 3 degrees. Non-isotropic voxels were obtained (0.6 × 0.6 × 0.8 mm), with a similar acquisition time of 5 min 4 s. 14 participants were recruited, but imaging could not be acquired in 2 participants due to non-compliance, and a further dataset was severely degraded by motion artefact. The age range for the available MRI datasets was 3 weeks to 9 years (median 1.8 years). Two examples of the 3D MRI rendering were provided, requiring an MRI processing time between 30 min and 2 h per patient. The long image processing time make this technique prohibitive. Fully automated segmentation techniques are a necessity for these techniques to finally be routinely adopted into clinical practice.

##### Head Trauma

Dremmen et al. [4] studied the imaging of 28 children with acute head trauma, where CT and MRI (with GRE-BB) imaging had been acquired within 7 days of each other. GRE-BB imaging showed a sensitivity of 66.7% and a specificity of 87.5% in the detection of skull fractures, utilising axial acquisition with reconstruction in the coronal and sagittal planes. Two false negative cases were misinterpreted as cranial sutures, and similarly two false positives also related to misinterpretation of sutures as fractures. A further false negative was due to misinterpretation of a fracture as a venous transosseous vascular channel. These cases highlight that with increasing use of the GRE-BB technique, familiarity with normal will be increased, and thus specificity improved. The one case where this would not improve was in a case where the fracture was in the mastoid region. Air and bone are almost impossible to discern on GRE-BB—one of the main limitations of the technique, which may be overcome by the addition of ZTE.

**Fig. 8:**
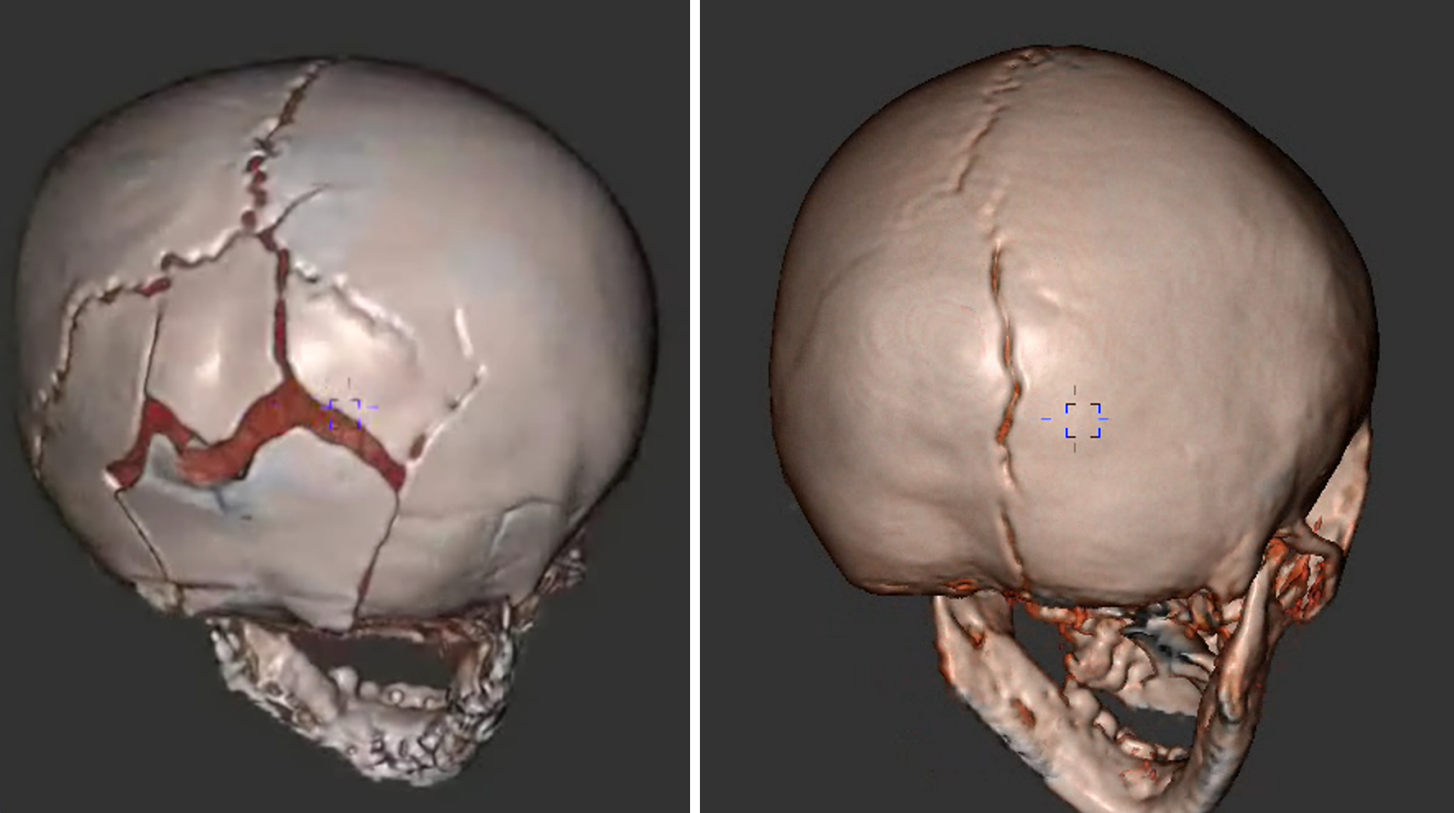
Semi-automated 3D segmentation of PETRA imaging in infants with skull fractures secondary to abusive head trauma using Fovia High-definition volume rendering software (MRI data acquisition courtesy of Dr S Kralik)

It is well recognised that the addition of 3D reconstructed images from CT increases the sensitivity and specificity for detecting skull fractures and is essential in the evaluation of paediatric head CTs for distinguishing subtle fractures from sutural variants, especially in the setting of trauma [32]. It is therefore unsurprising that the addition of 3D reconstructed MR images of the skull in children with abusive head trauma offered improved sensitivity and specificity compared to Dremmen et al. [4] study. PETRA imaging (ZTE) was successfully utilised in children between the ages of 1–30 months with semi-automated segmentation (Fig. 8). This yielded a sensitivity of 83% and specificity of 100% for skull fractures when compared to conventional CT imaging [19]. Similarly, in an adult population, Cho et al. [33] found that ZTE was equivalent to CT in the diagnosis of skull fracture in 13 patients, and that there was no significant difference in the measured skull thickness on both modalities.

##### Temporal Bone Imaging

MRI is typically the primary imaging modality in pre-operative cochlear implant assessments, providing the ability to assess cochlear nerve calibre and the patency of the cochlea. However, visualisation of bony anatomy, such as the mastoid facial nerve canal, are better served with CT. Connor et al. [34] were able to successfully delineate the mastoid facial nerve canal in all 25 adult patients, and the posterior canaliculus of the chorda tympani in 72% of cases. The authors concluded that performance was comparable to CT, and thus the addition of a GRE-BB technique would be valuable in identifying these landmarks and thus defining the facial recess.

##### Temporomandibular Joint Imaging

Lee et al. [35] evaluated the clinical usability of ZTE in assessing the bone changes of the temporomandibular joint, in comparison to CBCT. They found high reproducibility for ZTE, comparable to CBCT. The addition of a ZTE sequence to routine MRI examination would obviate additional CT imaging in this cohort.

##### Cephalometric Analysis

Cephalometric analysis was introduced in the early 1930’s and is a key element of orthodontic and orthognathic surgery. Traditionally measurements have been performed on two-dimensional radiographs. The limitation of this 2D representation of 3D anatomy led to increased use of CBCT or conventional CT. Justifying this additional radiation exposure is difficult.

The potential application of GRE-BB for cephalometric analysis was unveiled at the European Society of Head & Neck Radiology in 2010, and subsequently reported in the literature in 2013 [36]. This opened the door to potential radiation-free methods of 3D cephalometry. The main limitation of course remains that such analyses are frequently required in patients with fixed orthodontic appliances, for which a gradient echo sequence will result in significant metallic artefact. Subsequent research in this area has included the use of a T1-weighted 3D MSVAT-SPACE (multiple slab acquisition with view angle tilting gradient based on a sampling perfection with application optimized contrasts using different flip angle evolution) prototype sequence to perform 3D cephalometry [37].

##### Head & Neck Malignancy

**Fig. 9:**
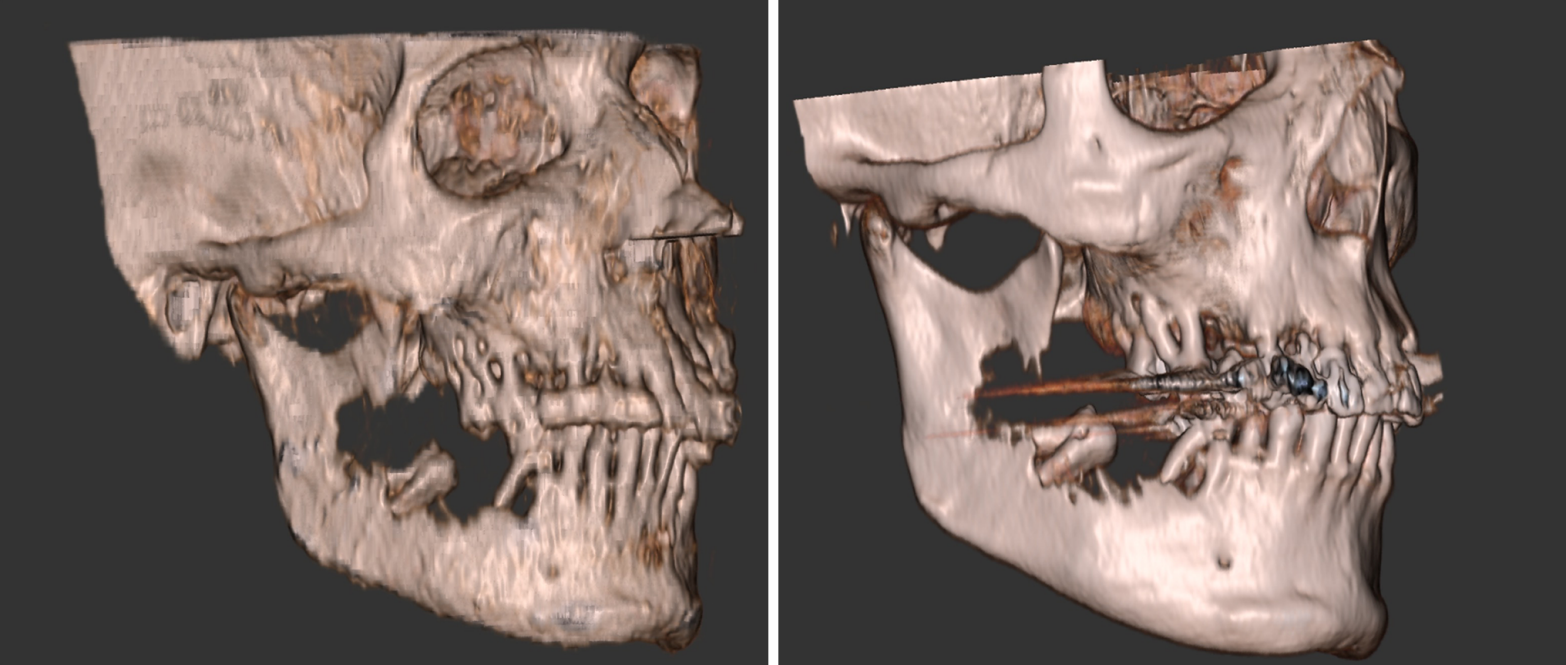
Manual 3D rendering of GRE-BB (left) demonstrating mandibular erosion and loose mandibular molar tooth, with comparable 3D CT (right)

**Fig. 10:**
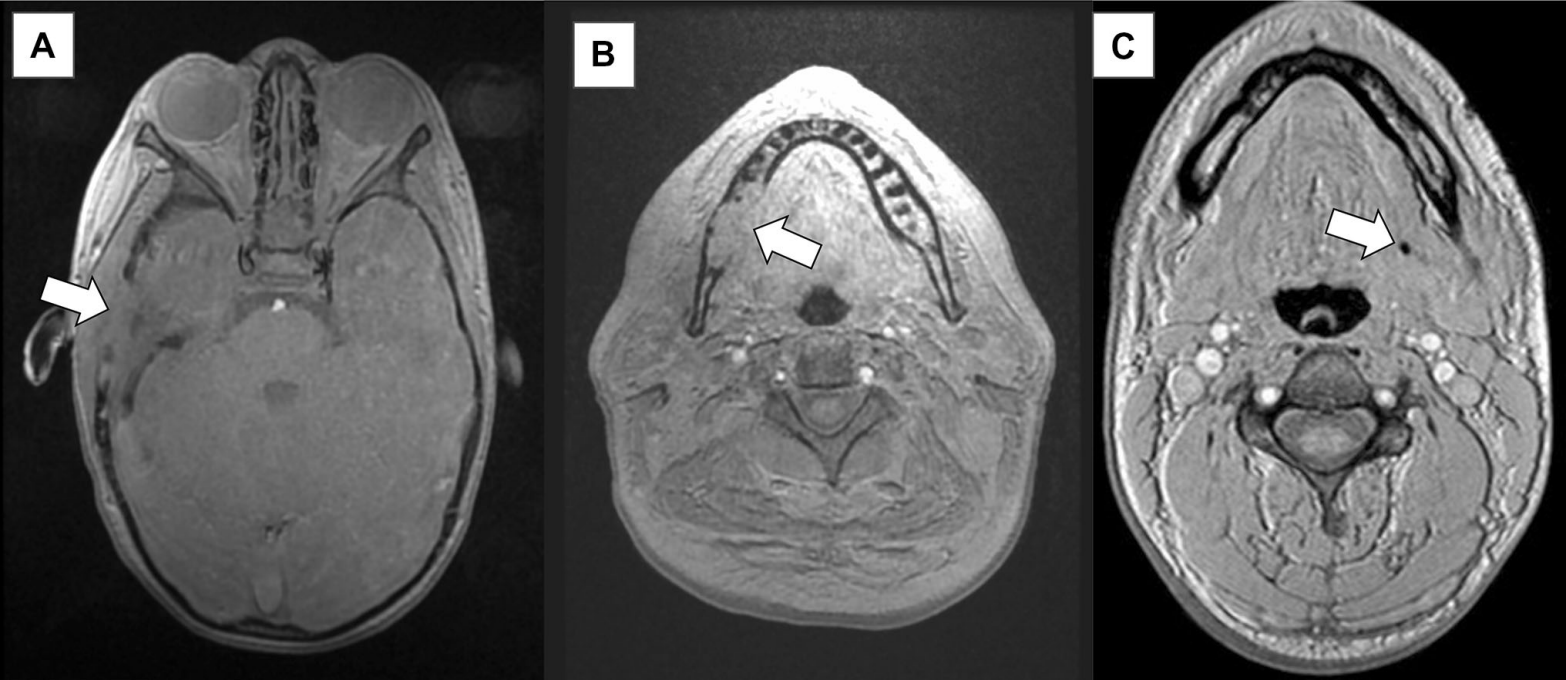
Range of pathology depicted on GRE-BB **A** child with right temporal bone defect due to Langerhans Cell Histiocytosis; **B** Right mandibular cortical erosion secondary to squamous cell carcinoma; **C** left submandibular duct calculus

MRI provides superior soft tissue imaging and delineation of tumours compared to CT. Staging of certain head and neck cancer subsites includes bone invasion as a reason for upstaging using the TNM system. Whilst marrow signal change is more easily detected on MRI, cortical erosion on standard sequences can be challenging. The addition of GRE-BB or ZTE permits improved visualisation of the cortical bone often obviating the need for CT imaging (Fig. 9 and 10). Since CT is frequently affected by dental amalgam artefact, this technique can be particularly valuable [38]. Suzuki et al. [39] evaluated 49 cases of suspected mandibular invasion comparing a conventional 2D FSE and high-resolution 3D VIBE with CT. All three techniques correctly diagnosed the 32 tumours with histopathological evidence of mandibular invasion (sensitivity 100%). The specificities of 2D FSE, 3D VIBE and CT were 56, 78 and 89%. It should be noted that the flip angle for VIBE was 20 degrees, and the TR/TE 13.9/3.9, and thus not sufficient to minimise the soft tissue contrast to produce Black Bone images. Further research in this area is required to determine if GRE-BB or ZTE can achieve a higher specificity for cortical erosion.

Tumour volume has previously been demonstrated to be a better predictor of survival in oral cavity cancers than linear measurements, with Black Bone being successfully utilised to perform volume measurements in a range of cases [40].

##### PET Attenuation Correction

Typical MRI-based attenuation correction methods for PET imaging use a fat/water map derived from a two-echo Dixon sequence, where bone is neglected. Leynes et al. [41] in 2017 proposed the use of patient-specific multi-parametric MRI consisting of Dixon MRI and proton-density weighted ZTE to directly synthesise pseudo-CT images with the use of a deep learning model. Wiesinger et al. [42] utilised proton density-weighted ZTE images to produce pseudo-CT which provided comparable attenuation information to CT. Delso et al. [43**] demonstrated excellent correlation between ZTE and CT bone segmentations, concluding that single-echo proton density-weighted ZTE is an efficient means of obtaining anatomically accurate maps of bone tissue for the purpose of PET attenuation correction.

##### Radiotherapy Planning

Treatment planning systems rely on electron density maps generated from CT images for calculating dose. Lei et al. [6] developed a learning-based method to generate patient specific synthetic CT from a routine anatomical MRI for use in radiotherapy planning. The algorithm was evaluated in 14 patient datasets consisting of MRI and CT, demonstrating the feasibility of MR based dose calculation.

##### Surgical Planning

Head and neck imaging is frequently acquired not for diagnostic purposes but for surgical planning of complex cases. Several authors have evaluated various MRI Black Bone techniques for this purpose. This includes CAD/CAM planning in children with non-syndromic metopic synostosis with successful production of cutting guides [44]. The authors also highlighted the added benefits of MRI acquisition in this patient cohort, namely superior illustration of the neurovascular and dural venous system, and brain anomalies [44].

Hoving et al. [45] developed a 3D virtual planning workflow based on GRE-BB for the lower jaw. The most challenging area to segment, as expected, was the coronoid process in view of the dense muscular attachment, but an area not of interest in most clinical settings.

3D printing is an integral component of surgical planning for a wide range of craniomaxillofacial surgical procedures. Once a 3D imaging dataset has been fully segmented, the process of creating a suitable file for 3D printing is relatively straight forward. The first 3D printed GRE-BB model was of an adult mandible created on a Z650 binder and powder 3D printer [46]. Utilising a cube phantom, it was confirmed that 3D printing from GRE-BB datasets demonstrated a similar level of accuracy to CT imaging datasets.

##### Other Applications

Recently, Low et al. [47] reported their experience of using GRE-BB for a range of head and neck pathologies in children. This included pathologies already discussed, and a range of focal osseous/fibro-osseous lesions such as fibrous dysplasia and Langerhans cell histiocytosis (LCH). Other reported applications include the assessment of facial asymmetry, benign tumours of the jaws, and facial fractures [48]. It can also assist in making salivary calculi more conspicuous (Fig. 10).

#### Limitations of MRI

The main limitation of MRI is the potential for motion artefact, which can be problematic in the paediatric population. However, in a recent study Jaimes et al. reported an overall success rate of 82% for non-sedated MRI examinations in children aged between 1–7 years [49]. As expected, young age impacted upon success. Feed and wrap methods are typically only successful in infants less than 3 months of age. However, Dean et al. [50] reported a success rate of 97% in children under 4 years of age undergoing MRI examination during natural non-sedated sleep. The use of silent ZTE techniques are particularly valuable when imaging children, and whilst GRE-BB is not a silent sequence, the noise that is generated is frequently well tolerated in view of its repetitive nature. MRI examinations are invariably more costly than CT, in view of the longer examination times. However, implementing these techniques in two cohorts of patients: (1) children with benign pathology, (2) patients already undergoing MRI and who would ordinarily require additional CT imaging, offers a cost-effective, efficient approach with radiation protection in the most vulnerable patient group.

## Conclusion

Recent developments in MRI technology and image processing provide the radiologist with a viable alternative to CT when imaging bone of the head and neck region. This is most valuable in children with benign pathology, and in all patients where the addition of bone-specific MR techniques could obviate the need for multi-modality imaging.

## References

[1] Hounsfield GN (1973). Computerized transverse axial scanning (tomography): Part I Description of system. Br J Radiol.

[2] Yuan MK, Tsai DC, Chang SC, Yuan MC, Chang SJ, Chen HW, Leu HB (2013). The risk of cataract associated with repeated head and neck CT studies: a nationwide population-based study. AJR Am J Roentgenol.

[3] Chen JX, Kachniarz B, Gilani S, Shin JJ (2014). Risk of malignancy associated with head and neck CT in children: a systematic review. Otolaryngol Head Neck Surg.

[4] Dremmen MHG, Wagner MW, Bosemani T, Tekes A, Agostino D, Day E, Soares BP, Huisman TAGM (2017). Does the Addition of a "Black Bone" Sequence to a Fast Multisequence Trauma MR Protocol Allow MRI to Replace CT after Traumatic Brain Injury in Children?. AJNR Am J Neuroradiol.

[5] Jeong DK, Lee SC, Huh KH (2012). Comparison of effective dose for imaging of mandible between multi-detector CT and cone-beam CT. Imaging Sci Dent.

[6] Lei Y, Harms J, Wang T, Tian S, Zhou J, Shu H-K, Zhong J, Mao H, Curran WJ, Liu T (2019). MRI-based synthetic CT generation using semantic random forest with iterative refinement. Phys Med Biol.

[7] Yang X, Wang T, Lei Y, Higgins K, Liu T, Shim H, Curran WJ, Mao H, Nye JA (2019). MRI-based attenuation correction for brain PET/MRI based on anatomic signature and machine learning. Phys Med Biol.

[8] Rozovsky K, Udjus K, Wilson N, Barrowman NJ, Simanovsky N, Miller E (2016). Cranial ultrasound as a first-line imaging examination for craniosynostosis. Pediatrics.

[9] Alizadeh H, Najmi N, Mehdizade M, Najmi N (2013). Diagnostic accuracy of ultrasonic examination in suspected craniosynostosis among infants. Indian Pediatr.

[10] Rabiner JE, Friedman LM, Khine H, Avner JR, Tsung JW (2013). Accuracy of point-of-care ultrasound for diagnosis of skull fractures in children. Pediatrics.

[11] Parri N, Crosby BJ, Mills L, Soucy Z, Musolino AM, Da Dalt L, Cirilli A, Grisotto L, Kuppermann N (2018). Point-of-care ultrasound for the diagnosis of skull fractures in children younger than two years of age. J Pediatr.

[12] Alexandridis G, Verschuuren EW, Rosendaal AV, Kanhai DA (2022). Evidence base for point-of-care ultrasound (POCUS) for diagnosis of skull fractures in children: a systematic review and meta-analysis. Emerg Med J.

[13] Gordon I, Sinert R, Chao J (2021). The utility of ultrasound in detecting skull fractures after pediatric blunt head trauma: systematic review and meta-analysis. Pediatr Emerg Care.

[14] Masaeli M, Chahardoli M, Azizi S (2019). Point of care ultrasound in detection of brain hemorrhage and skull fracture following pediatric head trauma; a diagnostic accuracy study. Arch Acad Emerg Med..

[15] Wiesinger F, Sacolick LI, Menini A, Kaushik SS, Ahn S, Veit-Haibach P, Delso G, Shanbhag DD (2016). Zero TE MR bone imaging in the head. Magn Reson Med.

[16] Robson MD, Gatehouse PD, Bydder M, Bydder G (2003). Magnetic resonance: an introduction to ultrashort TE (UTE) Imaging. J Comput Assisted Tomography.

[17] Eley KA, McIntyre AG, Watt-Smith SR, Golding SJ (2012). "Black bone" MRI: a partial flip angle technique for radiation reduction in craniofacial imaging. Br J Radiol.

[18] Eley KA, Watt-Smith SR, Sheerin F, Golding SJ (2014). "Black Bone" MRI: a potential alternative to CT with three-dimensional reconstruction of the craniofacial skeleton in the diagnosis of craniosynostosis. Eur Radiol.

[19] Kralik SF, Supakul N, Wu IC, Delso G, Radhakrishnan R, Ho CY, Eley KA (2019). Black bone MRI with 3D reconstruction for the detection of skull fractures in children with suspected abusive head trauma. Neuroradiology.

[20] Chang EY, Du J, Chung CB (2015). UTE imaging in the musculoskeletal system. J Magn Reson Imaging.

[21] Ljungberg E, Damestani NL, Wood TC, Lythgoe DJ, Zelaya F, Williams SCR, Solana AB, Barker GJ, Wiesinger F (2021). Silent zero TE MR neuroimaging: Current state-of-the-art and future directions. Prog Nucl Magn Reson Spectrosc.

[22] Fritz J (2021). Automated and radiation-free generation of synthetic CT from MRI Data: Does AI help to cross the finish line?. Radiology.

[23] Jans L, Chen M, Elewaut D (2021). MRI based synthetic CT in the detection of structural lesions in patients with suspected sacroiliitis: comparison with MRI. Radiology.

[24] Eley KA, Watt-Smith SR, Golding SJ (2011). Black bone MRI: three dimensional rendering and cephalometric analysis: an alternative to CT when imaging the craniofacial skeleton. Int J CARS.

[25] Eley KA, Watt-Smith SR, Golding SJ (2017). Three-dimensional reconstruction of the craniofacial skeleton with gradient echo magnetic resonance imaging ("Black Bone"): What is currently possible?. J Craniofac Surg.

[26] Eley KA, Delso G (2020). Automated segmentation of the craniofacial skeleton with “black bone” magnetic resonance imaging. J Craniofac Surg.

[27] Lu A, Gorny KR, Ho ML (2019). Zero TE MRI for craniofacial bone imaging. AJNR Am J Neuroradiol.

[28] Maeda J, Tanikawa C, Nagata N, Lim J, Kreiborg S, Murakami S, Yamashiro T (2021). Comparison of 3D mandibular surfaces generated by MRI and CT. Orthod Craniofac Res.

[29] Eley KA, Delso G (2021). Automated 3D MRI rendering of the craniofacial skeleton: using ZTE to drive the segmentation of black bone and FIESTA-C images. Neuroradiology.

[30] Hayashi T, Fujima N, Hamaguchi A, Masuzuka T, Hida K, Kodera S (2019). Non-invasive three-dimensional bone-vessel image fusion using black bone MRI based on FIESTA-C. Clin Radiol.

[31] Patel KB, Eldeniz C, Skolnick GB, Jammalamadaka U, Commean PK, Goyal MS, Smyth MD, An H. 3D pediatric cranial bone imaging using high-resolution MRI for visualizing cranial sutures: a pilot study, Journal of Neurosurgery: Pediatrics PED, 2020; 26(3), 311–317. Retrieved Aug 12, 2021, from https://thejns.org/pediatrics/view/journals/j-neurosurg-pediatr/26/3/article-p311.xml 10.3171/2020.4.PEDS20131 PMC7736460 32534502

[32] Purushothaman R, Desai S, Jayappa S, Choudhary AK, Ramakrishnaiah RH (2021). Utility of three-dimensional and reformatted head computed tomography images in the evaluation of pediatric abusive head trauma. Pediatr Radiol.

[33] Cho SB, Baek HJ, Ryu KH (2019). Clinical feasibility of zero TE Skull MRI in patients with head trauma in comparison with CT: A single-center study. AJNR Am J Neuroradiol.

[34] Connor SEJ, Borri M, Pai I, Barnsley H (2021). ‘Black Bone’ magnetic resonance imaging as a novel technique to aid the pre-operative planning of posterior tympanotomy for cochlear implantation. Cochlear Implants Int.

[35] Lee C, Jeon KJ, Han S-S, Kim YH, Choi YJ, Lee A (2020). CT-like MRI using the zero-TE technique for osseous changes of the TMJ. Dentomaxillofac Radiol.

[36] Eley KA, Watt-Smith SR, Golding SJ (2013). "Black Bone" MRI: a potential non-ionizing method for three-dimensional cephalometric analysis–a preliminary feasibility study. Dentomaxillofac Radiol.

[37] Juerchott A, Saleem MA, Hilgenfeld T (2018). 3D cephalometric analysis using magnetic resonance imaging: validation of accuracy and reproducibility. Sci Rep.

[38] Smith M, Bambach S, Selvaraj B, Ho M-L (2021). Zero-TE MRI: Potential applications in the oral cavity and oropharynx. Top Magn Reson Imaging.

[39] Suzuki N, Kuribayashi A, Sakamoto K, Sakamoto J, Nakamura S, watanabe H, Harada H, Kurabayashi T, (2019). Diagnostic abilities of 3T MRI for assessing mandibular invasion of squamous cell carcinoma in the oral cavity: comparison with 64-row multidetector CT. Dentomaxillofac Rad.

[40] Eley KA, Watt-Smith SR, Golding SJ (2013). Magnetic resonance imaging-based tumor volume measurements predict outcome in patients with squamous cell carcinoma of the mandible. Oral Surg Oral Med Oral Pathol Oral Radiol.

[41] Leynes AP, Yang J, Wiesinger F, Kaushik SS, Shanbhag DD, Seo Y, Hope TA, Larson PEZ (2017). Direct pseudoCT generation for pelvis PET/MRI attenuation correction using deep convolutional neural networks with multi-parametric MRI: zero echo time and Dixon deep pseudoCT (ZeDD-CT). JNumed.

[42] Wiesinger F, Bylund M, Yang J, Kaushik S, Shanbhag D, Ahn S, Jonsson JH, Lundman JA, Hope T, Nyholm T, Larson P, Cozzini C (2018). Zero TE-based pseudo-CT image conversion in the head and its application in PET/MR attenuation correction and MR-guided radiation therapy planning. Magn Reson Med.

[43] Delso G, Wiesinger F, Sacolick LI, Kaushik SS, Shanbhag DD, Hüllner M, Veit-Haibach P (2015). Clinical evaluation of zero-echo-time MR imaging for the segmentation of the skull. J Nucl Med.

[44] Lethaus B, Gruichev D, Gräfe D, Bartella AK, Hahnel S, Yovev T, Pausch NC, Krause M (2021). “Black bone”: the new backbone in CAD/CAM-assisted craniosynostosis surgery?. Acta Neurochir (Wien).

[45] Hoving AM, Kraeima J, Schepers RH, Dijkstra H, Potze JH, Dorgelo B, Witjes MJH (2018). Optimisation of three-dimensional lower jaw resection margin planning using a novel Black Bone magnetic resonance imaging protocol. PLoS ONE.

[46] Eley KA, Watt-Smith SR, Golding SJ (2017). "Black Bone" MRI: a novel imaging technique for 3D printing. Dentomaxillofac Radiol.

[47] Low XZ, Lim MC, Nga V, Sundar G, Tan AP (2021). Clinical application of "black bone" imaging in paediatric craniofacial disorders. Br J Radiol.

[48] Eley KA, Watt-Smith SR, Golding SJ (2012). "Black bone" MRI: a potential alternative to CT when imaging the head and neck: report of eight clinical cases and review of the Oxford experience. Br J Radiol.

[49] Jaimes C, Robson CD, Machado-Rivas F, Yang E, Mahan K, Bixby SD, Robertson RL (2021). Success of nonsedated neuroradiologic MRI in Children 1–7 Years Old. AJR Am J Roentgenol.

[50] Dean DC, Dirks H, O'Muircheartaigh J, Walker L, Jerskey BA, Lehman K, Han M, Waskiewicz N, Deoni SC (2014). Pediatric neuroimaging using magnetic resonance imaging during non-sedated sleep. Pediatr Radiol.

